# The NF‐κB transcription factor RelA directs mucosal‐associated invariant T‐cell development

**DOI:** 10.1111/imcb.70096

**Published:** 2026-02-23

**Authors:** Thomas S Fulford, Hui‐Fern Koay, Raelene Grumont, Darryl N Johnson, Sebastian Scheer, Hendrik J Nel, Ranjeny Thomas, Jeffrey YW Mak, David P Fairlie, Charis E Teh, Daniel HD Gray, Vanessa L Bryant, Colby Zaph, Lorraine A O'Reilly, Steven Gerondakis, Dale I Godfrey

**Affiliations:** ^1^ Department of Microbiology & Immunology at the Peter Doherty Institute for Infection and Immunity University of Melbourne Parkville VIC Australia; ^2^ Department of Biochemistry and Molecular Biology, Biomolecular Discovery Institute Monash University Clayton VIC Australia; ^3^ Frazer Institute University of Queensland Woolloongabba QLD Australia; ^4^ Institute for Molecular Bioscience University of Queensland Brisbane QLD Australia; ^5^ The Walter and Eliza Hall Institute of Medical Research Parkville VIC Australia; ^6^ Department of Medical Biology The University of Melbourne Melbourne VIC Australia; ^7^ Clinical Immunology and Allergy Department Royal Melbourne Hospital Melbourne VIC Australia; ^8^ Present address: Materials Characterization and Fabrication Platform, Department of Chemical Engineering University of Melbourne Parkville VIC Australia; ^9^ Present address: Department of Infection and Immunity Luxembourg Institute of Health Esch‐sur‐Alzette Luxembourg

**Keywords:** development, LUBAC, MAIT cells, NF‐κB, RelA

## Abstract

Mucosal‐associated invariant T (MAIT) cells are characterized by rapid responses to nonpeptide antigens via invariant T‐cell receptors (TCR), and expression of an “effector‐like” T‐cell phenotype. The transcription factor promyelocytic leukemia zinc finger (PLZF) is crucial for defining the function of MAIT cells and other unconventional T cells; however, the transcriptional programs that direct MAIT cell development are not fully elucidated. Here, we show that the canonical NF‐κB transcription factor RelA is critical for MAIT cell thymic development, but not responsiveness to antigen, whereas NF‐κB1 and c‐Rel make more limited contributions. MAIT cell development is also impaired in the absence of the linear ubiquitin signaling complex (LUBAC), an upstream regulator of NF‐κB signaling, implicating this pathway in establishing the MAIT cell pool. Collectively, these data suggest LUBAC and NF‐κB signals as elements of the transcriptional network controlling MAIT cell development.

## Graphical abstract

In this study, we found that the nuclear factor kappa light chain enhancer in activated B cells (NF‐κB) transcription factor RelA, and to a lesser extent NF‐κB1 and NF‐κB2, are key regulators of mucosal‐associated invariant T (MAIT)‐cell development and differentiation. We also found that ligases Hoip and Hoil of the linear ubiquitin chain assembly complex (LUBAC), a known regulator of NF‐κB signaling, are also critical for MAIT cell number. Together, these signaling pathways are an important part of the transcriptional program that controls MAIT cell thymic development.

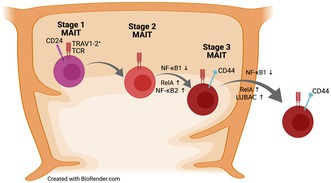

## INTRODUCTION

Mucosal‐associated invariant T (MAIT) cells comprise one of the largest antigen‐specific T‐cell populations. While MAIT cells are found in lymphoid and peripheral organs of both humans and mice, they are more abundant in human tissues.^1^, ^2^, ^3^ MAIT cells detect riboflavin (vitamin B2) metabolites presented by the MHC class I‐like molecule, MR1 on antigen‐presenting cells and use a semi‐invariant T‐cell receptor (TCR). In mice, the MAIT TCR consists of a TRAV1‐2/TRAJ33 α‐chain paired with a restricted repertoire of β‐chains.^4^ Similar to other unconventional T‐cell subsets, such as gamma‐delta (γδ) T cells and natural killer T (NKT) cells, MAIT cells develop in a three‐step process in the thymus.^5^, ^6^, ^7^, ^8^, ^9^ In mice, this pathway is defined by differential expression of CD24 and CD44 cell surface markers, with a CD24^+^CD44^−^ phenotype in the first stage, CD24^−^CD44^−^ during stage two, and CD24^−^CD44^+^ at the third stage.^9^ Step‐wise development depends on MAIT cells interacting with MR1 expressed by cortical CD4^+^CD8^+^ double‐positive thymocytes.^10^, ^11^, ^12^, ^13^ MR1 interacting with thymocytes not only defines the commitment to the MAIT lineage, but also maturation of the developing thymic MAIT cells.^9^, ^14^ The acquisition of a robust effector program is a defining feature of MAIT cell thymic development stages, underpinned by a transcriptional network dominated by promyelocytic leukemia zinc finger (PLZF), along with factors such as SATB1 and the SLAM adaptor SAP.^15^ The trajectory toward this effector phenotype can be discerned during the transition from Stage 2 to Stage 3. At Stage 3, functional diversification into T‐bet^+^ MAIT1 and RORγt^+^ MAIT17 subsets occurs in mice, but this is less defined in humans. While Stage 3 cells most closely resemble peripheral MAIT cells, further functional maturation occurs extrathymically.^9^

The NF‐κB family of transcription factors is part of a ubiquitous cell signaling pathway involved in controlling a diverse array of functions in immune cells. Canonical, or classical, NF‐κB transcription factors comprise homodimers or heterodimers of RelA (p65), c‐Rel and NF‐κB1 (or p105/p50); whereas the alternative, or noncanonical, pathway predominantly consists of NF‐κB2 (or p100/p52) homodimers or heterodimers with RelB.^16^ These homodimers and heterodimers interact with elements (κB motifs) in thousands of genes to positively and negatively regulate the expression of genes involved with processes as diverse as metabolism, differentiation and cell survival.^17^ This pathway is intimately involved with the development, differentiation and function of various lymphocyte subsets.^18^, ^19^ Notably, the canonical NF‐κB pathway plays a critical role in T‐cell subsets that have an activated or effector‐like phenotype, including during the thymic development and function of NKT cells, Treg cells and memory CD8 T cells.^20^, ^21^, ^22^, ^23^, ^24^, ^25^, ^26^ In contrast, the alternative pathway is linked with T‐cell‐extrinsic processes, including the development of thymic architecture and medullary thymic epithelial cell function, which nonetheless play a vital role in the development of T cells.^16^, ^19^ We and others have previously identified divergent transcriptional landscapes that control the development of MAIT cell populations, which showed an enrichment for NF‐κB regulated genes in Stage 3 MAIT cells.^15^, ^27^, ^28^ However, the transcriptional regulators that drive thymic development of MAIT cells remain poorly understood. Like NKT cells, MAIT cells exhibit a cell surface phenotype consistent with effector‐like T cells, including expression of CD44, CD161 and ICOS.^29^ Given the important role of NF‐κB proteins in the development and function of other unconventional and effector‐like T cells,^20^, ^21^, ^22^, ^23^, ^24^, ^25^, ^26^ we examined the impact of canonical and noncanonical NF‐κB family members on MAIT cell development using genetically deficient mice.

Here, we show that distinct members of the canonical NF‐κB family control MAIT cell development in a nonredundant manner. While the loss of c‐Rel did not have an apparent impact on MAIT cell development, NF‐κB1 deficient mice had increased MAIT cells, while RelA‐deficient mice had fewer MAIT cells. Absence of the alternative NF‐κB family member NF‐κB2 also leads to a decrease in developing MAIT cells. We also show that mice deficient in E3 ubiquitinases Hoip and Hoil, known regulators of NF‐κB signaling, also have impaired MAIT cell development, suggesting a role for the linear ubiquitin chain assembly complex (LUBAC) in MAIT cell development. Collectively, the NF‐κB family of transcription factors is critical regulators of MAIT cell thymic development, providing distinct signals to determine the size of the nascent MAIT cell pool.

## RESULTS

### NF‐κB family members differentially regulate thymic development of MAIT cells

**Figure 1:**
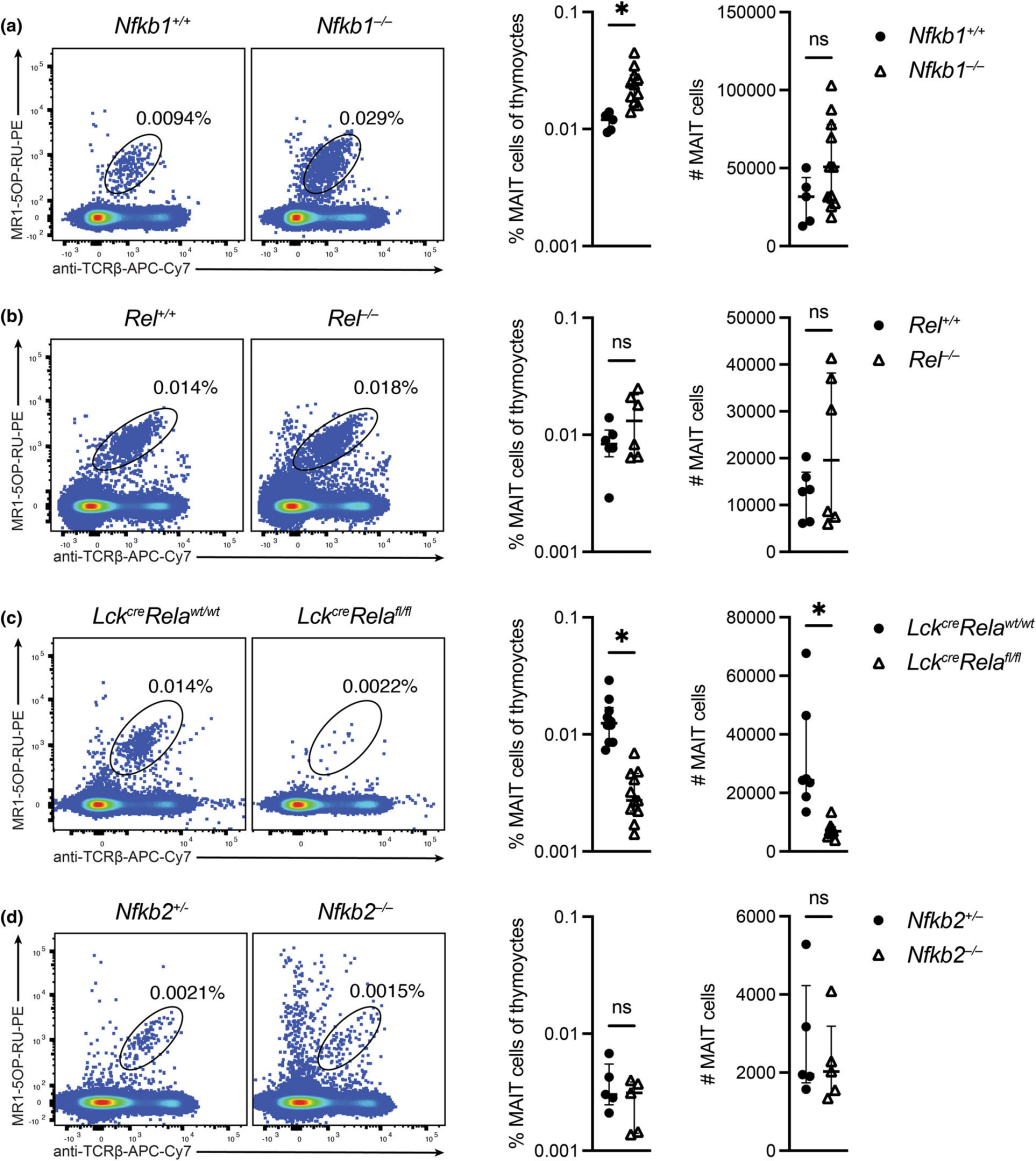
Thymic development of MAIT cells is regulated by NF‐κB signaling. Thymocytes from **(a)** *Nfkb1* ^*+/+*^ or *Nfkb1* ^*−/−*^, **(b)** *Rel* ^*+/+*^ or *Rel* ^*−/−*^, **(c)** *Lck* ^*cre*^ *Rela* ^*wt/wt*^ or *Lck* ^*cre*^ *Rela* ^*fl/fl*^, or **(d)** *Nfkb2* ^*+/−*^ or *Nfkb2* ^*−/−*^ mice were stained with MR1‐5‐OP‐RU tetramer‐PE and anti‐TCRβ‐APC‐Cy7 and analyzed for MAIT cell (*LHS*) frequency and (*RHS*) number by flow cytometry. Graphs depict median ± IQR. ns, nonsignificant; *P < 0.05 by the Mann–Whitney *U*‐test. *N* = 5 (*Nfkb1* ^*+/+*^, *Nfkb2* ^*+/+*^, *Nfkb2* ^*−/−*^), 11 (*Nfkb1* ^*−/−*^, *Rela* ^*fl/fl*^), 6 (*Rel* ^*+/+*^, *Rel* ^*−/−*^), 10 (*Rela* ^*wt/wt*^) biological replicates, except for *Rela* cell numbers where *n* = 7 (*Rela^wt/wt^*) or 8 (*Rela^fl/fl^*), from ≥ 2 experiments each. Each data point represents an individual biological replicate.

We examined the thymic development of MAIT cells in mice lacking canonical NF‐κB family members. NF‐κB1 is a nontranscriptionally active protein that nonetheless plays an important role in regulating NF‐κB signaling. The absence of NF‐κB1 in *Nfkb1* ^*−/−*^ mice led to a statistically significant increase in thymic MAIT cell frequency but not number (Figure 1a), suggesting NF‐κB1 plays a role in restraining normal MAIT cell development. MAIT numbers in *Nfkb1* ^*−/−*^ mice are likely not significantly elevated because of a slight decrease in overall thymic cellularity. The loss of c‐Rel had no appreciable impact on MAIT cell development (Figure 1b), whereas RelA was critical for thymic MAIT cell ontogeny by both frequency and number (Figure 1c). The latter was determined using mice that lack RelA specifically in T lineage cells by utilizing the LoxP‐Cre system (*Lck* ^*cre*^ *Rela* ^*fl/fl*^) (Figure 1c). In line with previous studies,^23^, ^30^ NF‐κB1‐deficient mice had a moderate but not significant reduction in mean frequency of NKT cells and a significant decrease in NKT cell numbers (Supplementary figure 1a), and a reduction in RelA T‐cell‐deficient mice (Supplementary figure 1c). In contrast to previous reports, mice lacking c‐Rel demonstrated a modest but significant decrease in NKT cell frequency, although this observation did not extend to cell numbers (Supplementary figure 1b).

**Figure 2:**
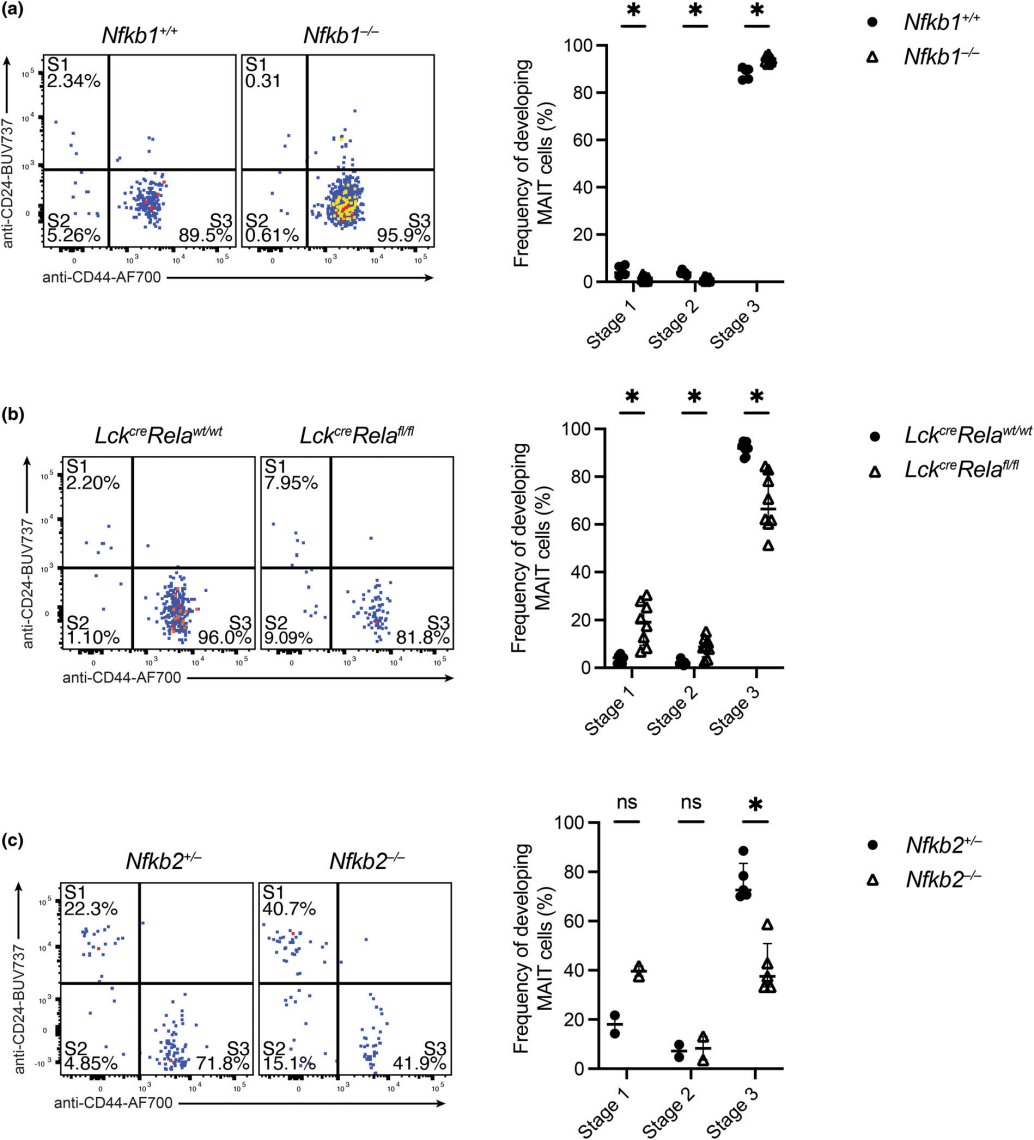
The lack of RelA or NF‐κB2 leads to a fewer mature MAIT cells in the thymus. Developing MAIT cells from the thymus of **(a)** *Nfkb1* ^*+/+*^ or *Nfkb1* ^*−/−*^, **(b)** *Lck* ^*cre*^ *Rela* ^*wt/wt*^ or *Lck* ^*cre*^ *Rela* ^*fl/fl*^ or **(c)** *Nfkb2* ^*+/−*^ or *Nfkb2* ^*−/−*^ mice were stained with anti‐CD24‐BUV737 and anti‐CD44‐AF700 and analyzed for MAIT cell developmental stage by flow cytometry. Graphs depict median ± IQR. ns, nonsignificant; S1, stage 1 [CD24^+^CD44^−^]; S2, stage 2 [CD24^−^CD44^−^]; S3, stage 3 [CD24^−^CD44^+^]; *P < 0.05 by the Mann–Whitney *U*‐test. *N* = 5 (*Nfkb1* ^*+/+*^, *Nfkb2* ^*+/+*^, *Nfkb2* ^*−/−*^), 11 (*Nfkb1* ^*−/−*^), 7 (*Rela* ^*wt/wt*^) or 8 (*Rela* ^*fl/fl*^) biological replicates from ≥ 2 experiments each, except for discrimination of stage 1 and stage 2 in one *Nfkb2* experiment, which could not be identified because of the lack of anti‐CD24 in that experiment (*n* = 2, 1 experiment). Each data point represents an individual biological replicate.

We have previously described three developmental stages of MAIT cell development that can be identified by the differential expression of CD24 and CD44.^9^ Utilizing the same strategy, we demonstrate that expansion of MAIT cells in *Nfkb1* ^*−/−*^ mice corresponded with a subtle, but significant, expansion of mature stage (S)3 (CD24^−^CD44^+^) MAIT cells compared with the other two developmental stages (Figure 2a). Conversely, the reduction of MAIT cells in the absence of RelA correlated with fewer MAIT cells reaching S3 and a corresponding increase in S1 (CD24^+^CD44^−^) and S2 (CD24^−^CD44^−^; Figure 2b). While this decrease is significant, it does not seem to account for the total reduction in MAIT cell numbers. Thus, the absence of RelA leads to an overall reduction in MAIT cells as well as reduced maturation of MAIT cells in the thymus.

We also examined mice lacking components of the alternative NF‐κB pathway, which also suggested a role in MAIT cell development. The absolute percentage and number of MAIT cells was not significantly altered in the thymus of *Nfkb2* ^*−/−*^ mice (Figure 1d); although the frequency of mature S3 MAIT cells in these mice was lower (Figure 2c). S1 and S2 MAIT cells were only examined in one experiment with the *Nfkb2* ^−/−^ mice because of the antibody combinations available at the time. As previously reported,^31^ NKT cells were reduced in *Nfkb2* ^*−/−*^ mice (Supplementary figure 1d). The alternative pathway regulates the development of thymic stromal architecture and medullary thymic epithelial cells (mTEC) function, with a putative role during NKT cell development in thymic stromal cells.^16^, ^30^ Thus, impaired MAIT cell development in NF‐κB2‐deficient mice likely reflects MAIT cell–extrinsic, stromal defects. Notably, RelA and NF‐κB2 can form functional heterodimers,^32^ raising the possibility that the MAIT cell developmental phenotype observed in NF‐κB2‐deficient mice may also involve cell‐intrinsic disruption of RelA–NF‐κB2 complexes.

### Mature MAIT cells maintain a requirement for NF‐κB proteins in the periphery

**Figure 3:**
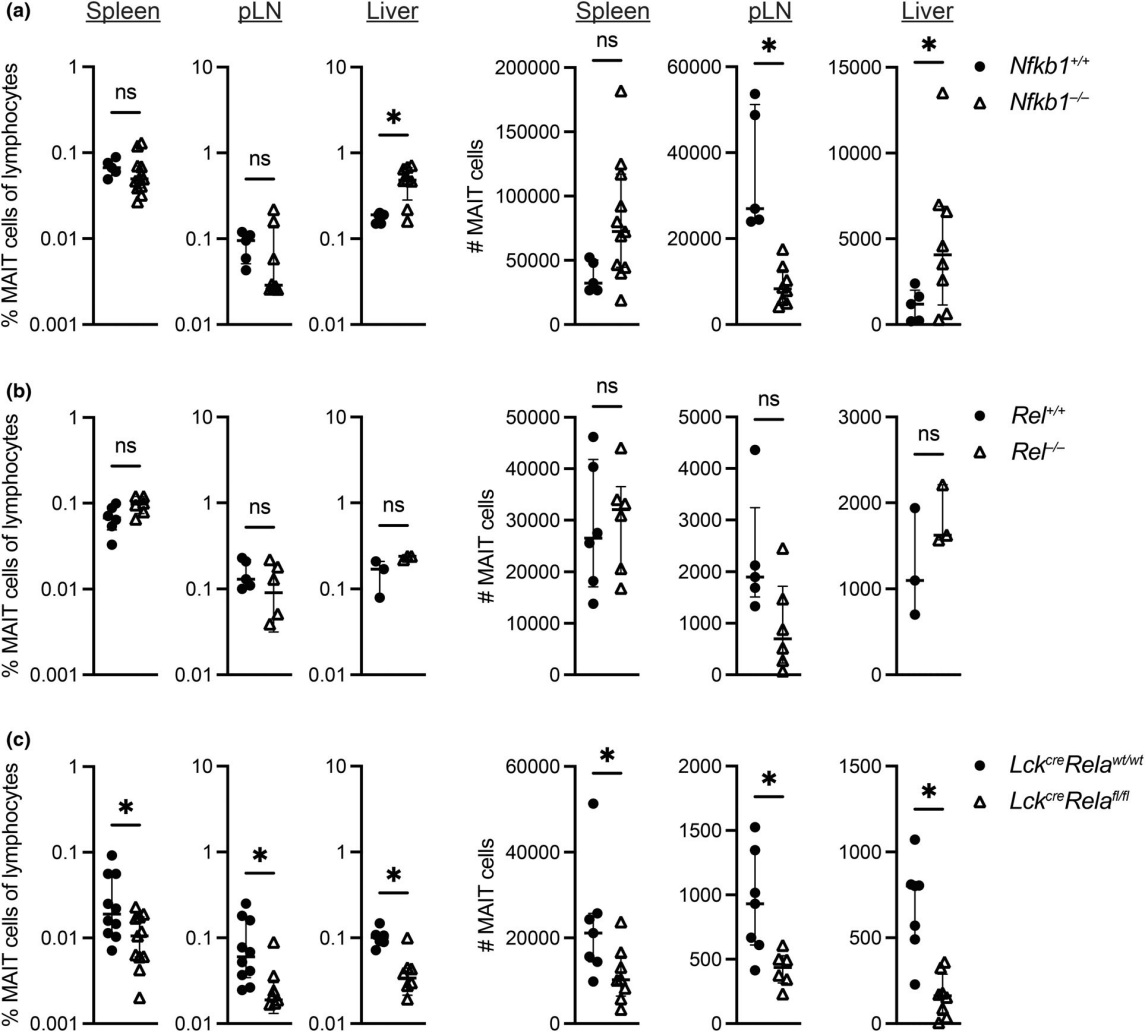
MAIT cells lacking RelA are unable to fill the peripheral niche. Lymphocytes from the spleen, peripheral lymph node (pLN) and liver of **(a)** *Nfkb1* ^*+/+*^ or *Nfkb1* ^*−/−*^, **(b)** *Rel* ^*+/+*^ or *Rel* ^*−/−*^ or **(c)** *Lck* ^*cre*^ *Rela* ^*wt/wt*^ or *Lck* ^*cre*^ *Rela* ^*fl/fl*^ mice were stained with MR1‐5‐OP‐RU tetramer‐PE and anti‐TCRβ‐APC‐Cy7 and analyzed for MAIT cell (*LHS*) frequency and (*RHS*) number by flow cytometry. Graphs depict median ± IQR. ns, nonsignificant; *P < 0.05 by the Mann–Whitney *U*‐test. For % graphs, *n* = 5 (*Nfkb1^+/+^* all tissues, *Rel^+/+^* pLN), 11 (*Nfkb1^–/–^* spleen, *Rela^fl/fl^* spleen), 8 (*Nfkb1^–/–^* pLN and liver, *Rela^fl/fl^* liver), 7 (*Rela^wt/wt^* liver), 6 (*Rel^+/+^* spleen, *Rel^–/–^* spleen and pLN), 10 (*Rela^wt/wt^* spleen and pLN), 9 (*Rela^fl/fl^* pLN), 3 (*Rel^+/+^* and *Rel^–/–^* liver). For # graphs, *n* is the same as % graphs except for *Rela^wt/wt^* spleen, pLN and liver (*n* = 7), *Rela^fl/fl^* pLN (*n* = 6), spleen and liver (*n* = 8). Biological replicates from ≥ 2 experiments, except *Rel^+/+^* and *Rel^–/–^* liver (1 experiment). Each data point represents an individual biological replicate.

Following selection in the thymus, MAIT cells migrate to peripheral tissues, such as liver and peripheral lymphoid tissues. NF‐κB1 and c‐Rel were largely dispensable for MAIT cell maintenance in secondary lymphoid organs (Figure 3a, b), although there was a moderate, albeit significant, increase in frequency of NF‐κB1‐deficient MAIT cells in liver, corresponding with their increase in thymus (Figure 3a). Consistent with the thymic data, the frequencies of MAIT cells in spleen, LN and liver of *Lck* ^*cre*^ *Rela* ^*fl/fl*^ mice were lower than *Lck* ^*cre*^ *Rela* ^*wt/wt*^ littermates (Figure 3c). While reduced peripheral MAIT cell frequencies in *Lck* ^*cre*^ *Rela* ^*fl/fl*^ mice suggest a diminished capacity to populate the peripheral niche, this could reflect impaired thymic output rather than a defect in niche‐filling competence *per se*. This distinction is further clarified by hematopoietic stem cell chimeras reconstituted with RelA‐deficient fetal liver cells (*Rela* ^*−/−*^ HSC chimeras) having diminished peripheral MAIT cell frequencies compared with controls reconstituted with wild‐type fetal liver cells, analogous to the reduction observed in NKT cells (Supplementary figure 2a and b). The equivalent frequency of MAIT cells in the thymus of *Rela* ^*−/−*^ HSC chimeras likely reflects the number of MAIT cells following radioablation being at the limit of detection by flow cytometry, at a given snapshot in time. Whereas the reductions observed in the peripheral tissues are because of the accumulated total thymic output following reconstitution, as well as potential defects in filling the peripheral niche. Overall, these data support a T‐cell‐intrinsic requirement for RelA in MAIT cell development, although we cannot rule out a supporting role for RelA in CD4^+^CD8^+^ double‐positive thymocyte‐mediated selection of MAIT cells. Once in the periphery, it also appears that mature RelA‐deficient MAIT cells have limited capacity to expand and fill the MAIT cell niche.

### MAIT cell maturation is regulated by NF‐κB RelA

**Figure 4:**
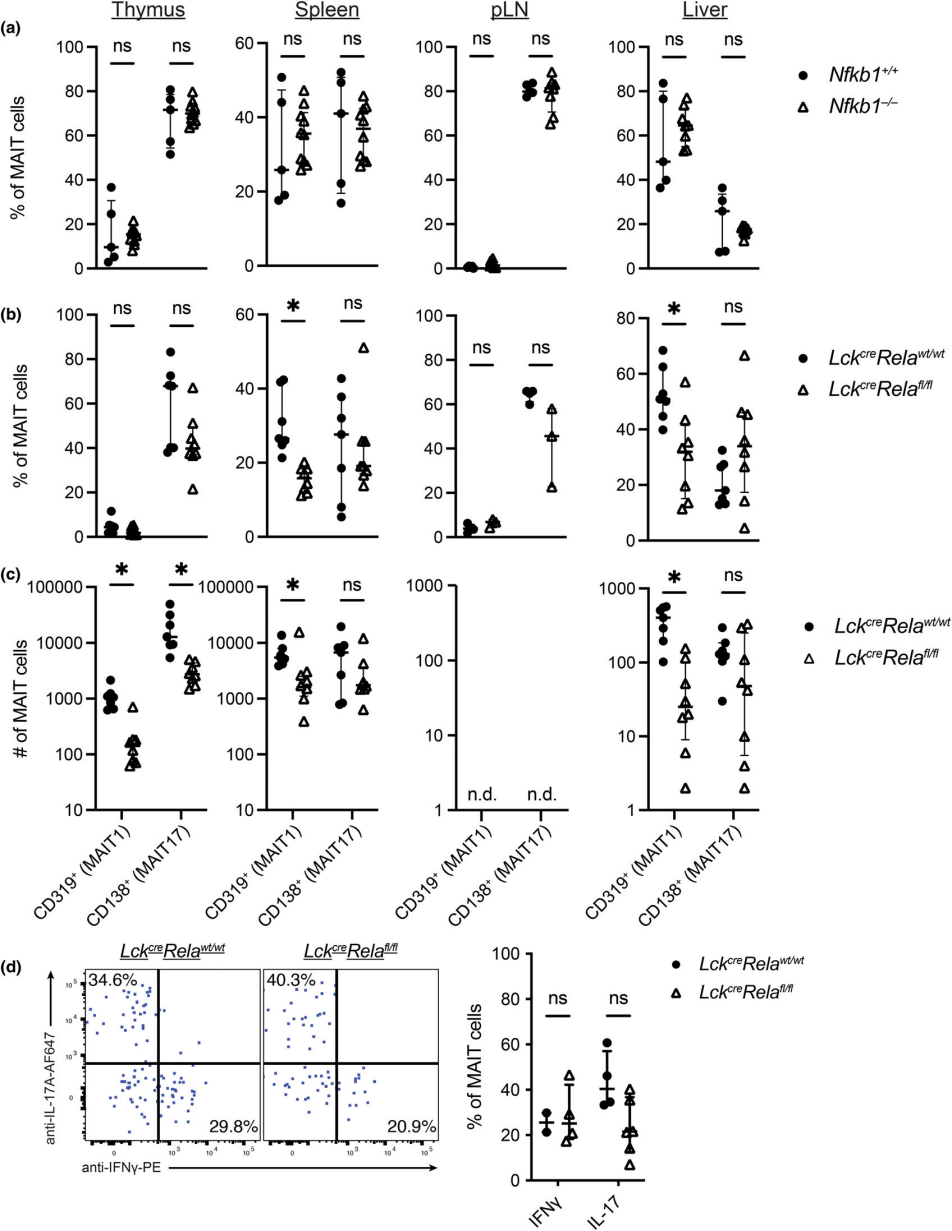
RelA is more important for the maturation of CD319^+^ MAIT1 cells than CD138^+^ MAIT17. MAIT cells from the thymus, spleen, peripheral lymph node (pLN) and liver of **(a)** *Nfkb1* ^*+/+*^ or *Nfkb1* ^*–/–*^ or **(b,c)** *Lck* ^*cre*^ *Rela* ^*wt/wt*^ or *Lck* ^*cre*^ *Rela* ^*fl/fl*^ mice were stained with anti‐CD138‐BB515 and anti‐CD319‐APC and analysed for MAIT cell phenotype by flow cytometry, as per ^15^. *N* = 5 (*Nfkb1* ^*+/+*^ all tissues), 10 (*Nfkb1* ^*–/–*^ thymus and spleen), 7 (*Rela* ^*wt/wt*^ thymus, spleen and liver), 8 (*Rela* ^*fl/fl*^ thymus, spleen and liver, *Nfkb1* ^*–/–*^ pLN and liver), 3 (*Rela* ^*wt/wt*^ and *Rela* ^*fl/fl*^ pLN) biological replicates from ≥ 2 experiments each, except *Rela* pLN (*n* = 3, 1 experiment). **(d)** Splenocytes from *Lck* ^*cre*^ *Rela* ^*wt/wt*^ or *Lck* ^*cre*^ *Rela* ^*fl/fl*^ mice were stimulated with PMA and Ionomycin for 4 hrs in the presence of GolgiStop. Expression of IFNγ and IL‐17A by MAIT cells was analysed by flow cytometry. *N* = 2 (*Rela* ^*wt/wt*^ IFNγ), 4 (*Rela* ^*fl/fl*^ IFNγ, *Rela* ^*wt/wt*^ IL‐17), or 6 (*Rela* ^*fl/fl*^ IL‐17) biological replicates from 1 (IFNγ) or 2 (IL‐17) experiments. Graphs depict median ± IQR. n.d. – no data; ns – non‐significant. *P < 0.05 by the Mann‐Whitney *U‐*test. Each data point represents an individual biological replicate.

In addition to the three‐stage thymic development pathway, MAIT cells undergo functional differentiation into T‐bet^+^ MAIT1 and RORγt^+^ MAIT17 cells.^15^, ^29^, ^33^, ^34^ Using Slamf7 (CD319) and CD138 as surrogate markers of these subsets, respectively,^15^ we examined MAIT1 and MAIT17 sub‐populations. NF‐κB1 was dispensable for the differentiation of MAIT cells into MAIT1 or MAIT17, as defined by the equivalent frequencies of CD319^+^ and CD138^+^ MAIT cells in *Nfkb1* ^*+/+*^ and *Nfkb1* ^*−/−*^ mice (Figure 4a). In contrast, RelA appears to be mainly involved in maintenance of CD319^+^ (MAIT1) cells in peripheral tissues (Figure 4b). The frequencies in spleen and liver (Figure 4b) and total numbers in thymus, spleen and liver (Figure 4c) of CD319^+^ MAIT1 cells were diminished in RelA‐deficient mice, whereas the CD138^+^ MAIT17 cell compartment remained largely unaffected, aside from a reduction in the number of these cells in thymus. Despite this apparent skewing of MAIT1/MAIT17 differentiation, as defined by CD319 and CD138 surrogate marker expression, splenic MAIT cells from *Lck* ^*cre*^ *Rela* ^*fl/fl*^ mice retained the capacity to produce IFNγ or IL‐17A upon stimulation with PMA and ionomycin (Figure 4d). This suggests that RelA is important for MAIT cell subset differentiation and maturation, but not necessarily for effector cytokine production post‐thymically.

### The absence of RelA does not prevent antigen‐specific activation and expansion of MAIT cells

**Figure 5:**
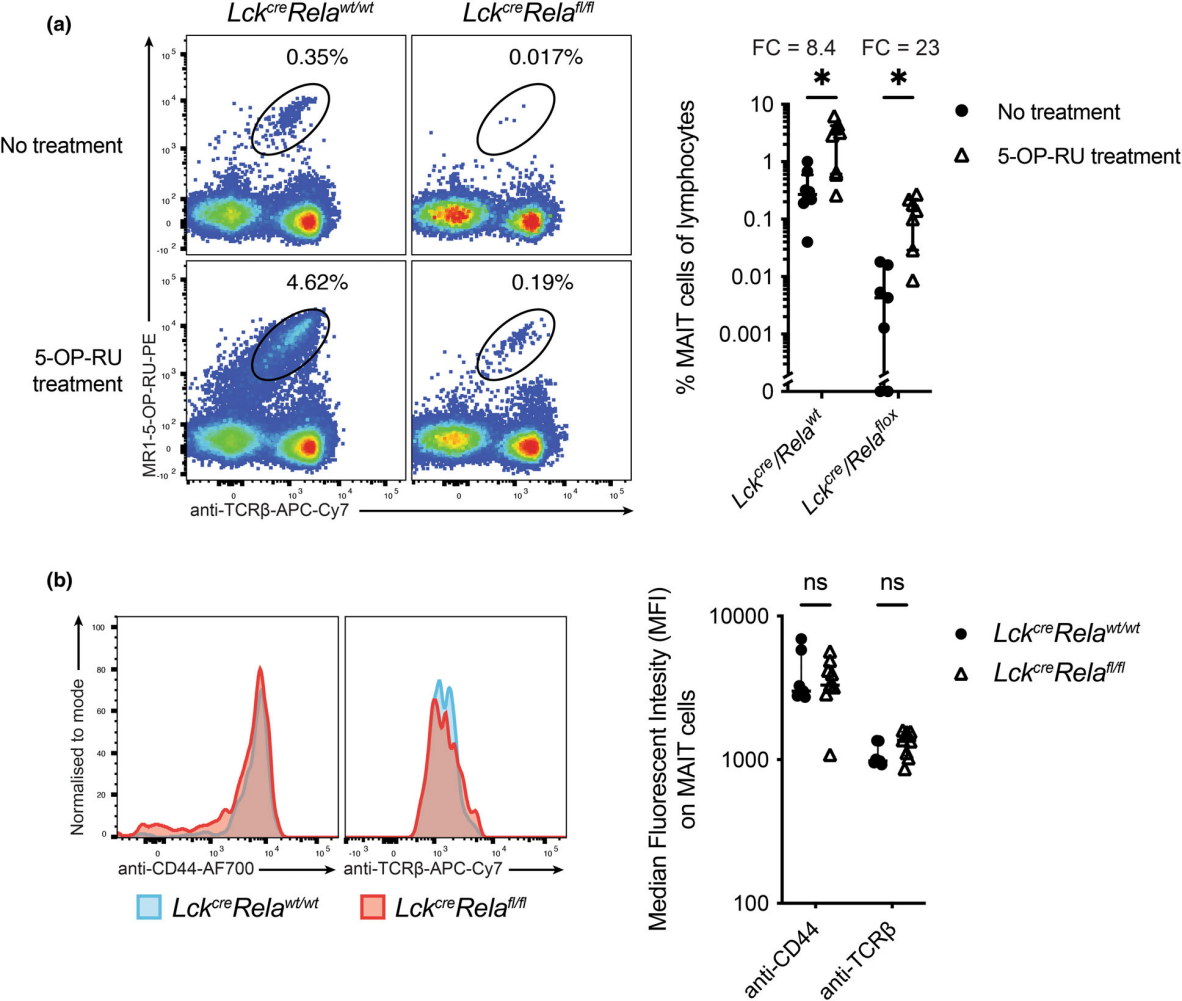
RelA‐deficient MAIT cells still respond to antigenic stimulation. **(a)** Splenocytes from *Lck* ^*cre*^ *Rela* ^*wt/wt*^ or *Lck* ^*cre*^ *Rela* ^*fl/fl*^ mice were cultured in the presence or absence of the MAIT cell antigen 5‐OP‐RU for 7 days. Cells were stained with MR1‐5‐OP‐RU tetramer‐PE and anti‐TCRβ‐APC‐Cy7 and analyzed for MAIT cell frequency following stimulation. Graphs depict median ± IQR. *P < 0.05 by Wilcoxon matched pairs signed rank test with Holm‐Šidák correction for multiple comparisons. *N* = 7 (*Rela^fl/fl^*) or 8 (*Rela^wt/wt^*) biological replicates from two experiments. Each data point represents an individual biological replicate. FC – fold change in MAIT cell frequency in 5‐OP‐RU treated compared with untreated. **(b)** Splenic MAIT cells from *Lck* ^*cre*^ *Rela* ^*wt/wt*^ or *Lck* ^*cre*^ *Rela* ^*fl/fl*^ mice were stained with anti‐CD44‐AF700 and anti‐TCRβ‐APC‐Cy7, and expression levels analyzed by flow cytometry. ns – nonsignificant by the Mann–Whitney *U*‐test. *N* = 7 (*Rela* ^*wt/wt*^), 9 (*Rela* ^*fl/fl*^) biological replicates from three experiments. Each data point represents an individual biological replicate.

Given the pronounced reduction of MAIT cells in both *Lck* ^*cre*^ *Rela* ^*fl/fl*^ mice and *Rela* ^*−/−*^ HSC chimeras, we next examined whether RelA‐deficient MAIT cells retained the capacity to respond to antigenic stimulation. Splenocytes isolated from *Lck* ^*cre*^ *Rela* ^*wt/wt*^ and *Lck* ^*cre*^ *Rela* ^*fl/fl*^ mice were incubated in the presence or absence of the cognate antigen 5‐OP‐RU for 7 days. Despite lower baseline frequencies, the average expansion (fold change) of MAIT cells from *Lck* ^*cre*^ *Rela* ^*fl/fl*^ mice was 23‐fold following stimulation, compared with approximately eightfold in controls (Figure 5a). Nevertheless, there is still a greater proportion of MAIT cells from 5‐OP‐RU stimulated *Lck* ^*cre*^ *Rela* ^*wt/wt*^ spleen than from *Lck* ^*cre*^ *Rela* ^*fl/fl*^ spleen, even accounting for the enhanced expansion of RelA‐deficient MAIT cells. Additionally, markers of TCR engagement, such as CD44 expression or TCRβ downregulation, were comparable between these groups (Figure 5b). These data suggest that the ability of MAIT cells to respond to cognate antigen is not RelA‐dependent, and by inference, that the altered maturation of MAIT cells in the absence of RelA may not be because of defective TCR responses.

### T‐cell deletion of the E3 ubiquitin ligases Hoil and Hoip, but not the adaptor Sharpin, reduces MAIT cell frequencies

**Figure 6:**
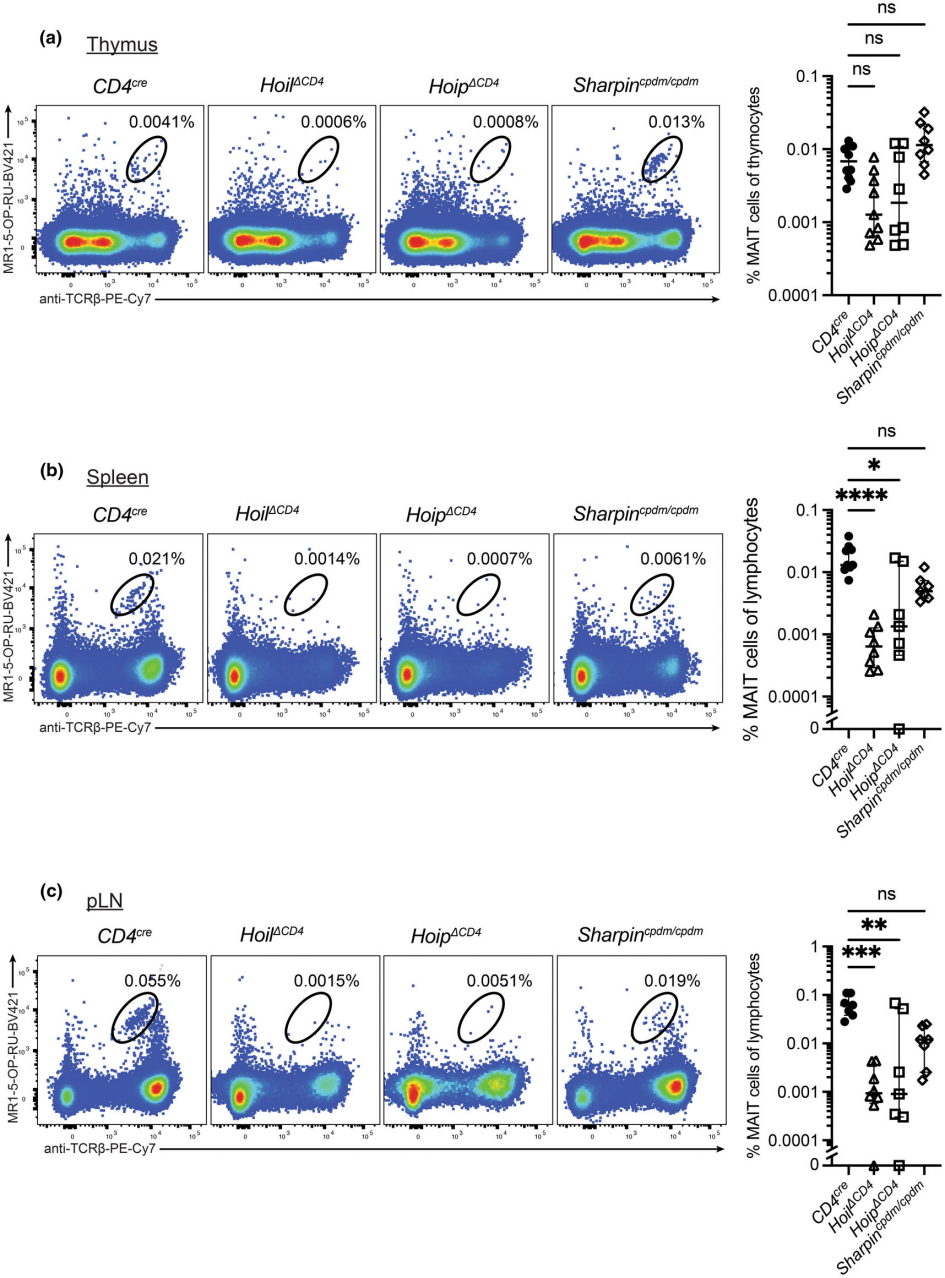
Mice deficient in LUBAC signaling have reduced MAIT cell frequencies. Lymphocytes from the **(a)** thymus, **(b)** spleen and **(c)** peripheral lymph node (pLN) of control *CD4* ^*cre*^ or *Hoip* ^*ΔCD4*^, *Hoil* ^*ΔCD4*^ or *Sharpin* ^*cpdm/cpdm*^ mice were stained with MR1‐5‐OP‐RU tetramer‐BV421 and anti‐TCRβ‐PE‐Cy7 and analyzed for MAIT cell frequency by flow cytometry. Graphs depict median ± IQR. ns, nonsignificant; *P < 0.05; **P < 0.01; ***P < 0.001; ****P < 0.0001 by Kruskal–Wallis test with Dunn's correction for multiple comparisons. *N* = 10 (*CD4* ^*cre*^ thymus), 9 (*Hoil* ^*ΔCD4*^ thymus, *CD4* ^*cre*^ spleen) or 8 (*Hoip* ^*ΔCD4*^ and *Sharpin* ^*cpdm/cpdm*^ thymus, *Hoil* ^*ΔCD4*^ spleen and pLN), 7 (*CD4* ^*cre*^ pLN, *Hoip* ^*ΔCD4*^ and *Sharpin* ^*cpdm/cpdm*^ spleen and pLN) biological replicates from two experiments. Each data point represents an individual biological replicate.

The linear ubiquitin chain assembly complex (LUBAC) comprising the two E3 ubiquitin ligases, Hoil and Hoip, along with the adaptor protein Sharpin, mediates M1‐linked linear ubiquitination,^35^ and is an upstream regulator of canonical NF‐κB signaling via linear ubiquitination of NEMO.^36^, ^37^ We examined the role of LUBAC in MAIT cell development using mice harboring a specific deletion of *Hoip* or *Hoil* in T cells (*Cd4* ^*cre*^ *Hoip* ^*fl/fl*^, here called *Hoip* ^*ΔCD4*^, and *Cd4* ^*cre*^ *Hoil1* ^*fl/fl*^, here *Hoil* ^*ΔCD4*^, respectively), or mice with the chronic proliferative dermatitis (*cpdm*) mutation that causes an early termination of *Sharpin* (*Sharpin* ^*cpdm/cpdm*^). The lack of either Hoip or Hoil led to impaired MAIT cell frequencies in periphery and reduced, albeit not significant, thymic MAIT cell development. In contrast, the loss‐of‐function *Sharpin* ^*cpdm/cpdm*^ mutation had no significant impact on thymic MAIT cell development or peripheral MAIT cell numbers (Figure 6a–c). Taken together, these data suggest that Hoil and Hoip play pivotal roles in MAIT cell maturation and function, although the loss‐of‐function of the adaptor protein Sharpin that forms a complex with Hoip and Hoil is less penetrant.

In summary, defects in transcription mediated by RelA or NF‐κB2, or upstream signaling through the E3 linear ubiquitination ligases Hoip and Hoil, lead to impaired MAIT cell development, particularly during the transition from S2 to S3, whereas loss of NF‐κB1 increases MAIT cell development.

## DISCUSSION

The NF‐κB pathway is involved in the development and function of several immune cell types including unconventional T cells. We and others have shown previously that NKT cells are dependent on RelA for development and function,^23^, ^24^, ^25^, ^30^, ^38^ whereas γδ T cells exhibit limited dependence on NF‐κB signaling for development, with a specific reliance on RelA for maturation of the IL‐17‐producing γδ17 subset.^39^ Similar NF‐κB dependencies are observed in effector‐like populations, including regulatory T cells and cytokine‐producing effector T cells (including IL‐2, GM‐CSF and IFNγ),^40^, ^41^ as well as germinal center B cell and plasma cell differentiation.^42^

Our findings here extend this paradigm to MAIT cells, revealing distinct roles for individual NF‐κB family members. NF‐κB1, which is typically considered a negative regulator of NF‐κB signaling in its homodimeric form, seems to have divergent roles in NKT and MAIT cell development. While the lack of NF‐κB1 has previously been shown to result in reduced NKT cell development.^23^, ^24^, ^25^, ^30^, ^38^ in contrast, we observed increased MAIT cell development in the absence of this factor. While the median percentage of NKT cells in the thymus in our study was moderately lower, in line with previous studies, this did not reach statistical significance; but nonetheless, this highlights a difference in the role of NF‐κB1 in regulating MAIT cells versus NKT cells. These divergent effects suggest T lineage‐specific functions of NF‐κB1, potentially mediated by differential interactions with transcriptional co‐regulators such as CREB‐binding protein (CBP)^43^ in NKT cells to regulate transcription positively, but as a transcriptional inhibitor in MAIT cells, implying context‐specific roles for different NF‐κB family members in unconventional T cells. This also may partially explain the divergence between mice lacking RelA in T cells and *Nfkb1* ^*−/−*^ mice, both key members of the “canonical” pathway. The additional binding partners of NF‐κB1 preclude direct attribution of “NF‐κB1‐specific” transcriptional landscape in MAIT cell development *per se*. Interestingly, we have demonstrated previously that a lack of developing NKT cells leads to an expansion of MAIT cells, potentially via increased accessibility to a common niche or survival factor(s).^9^, ^44^ Thus, the modest increase in MAIT cell frequency in the thymus of *Nfkb1* ^*−/−*^ mice may be because of a loss of NKT cells and a corresponding increased access to a shared unconventional T‐cell niche, rather than an intrinsic role for NF‐κB1 in restraining MAIT cell development.

Consistent with reports investigating NKT cells,^23^ c‐Rel is largely dispensable for MAIT cell development, although surprisingly in our study, we did find a small but significant reduction in NKT cell development in mice lacking c‐Rel. The loss of NF‐κB2 also has a negative impact on MAIT cell development, although the lack of available mice limits the power of the current observations. Traditionally, the alternative NF‐κB pathway has been associated with lymphoid organ architecture and stromal cell development.^16^ Reports have confirmed a role for the alternate pathway supporting stromal cell function in NKT cell development.^30^, ^45^ Curiously, T‐cell intrinsic roles for components of the alternative pathway have also been identified in Th17 cell^46^ and other unconventional T‐cell development.^39^ While the role of NF‐κB2 in MAIT cell development is likely to be via supporting stromal cells, this remains to be shown experimentally.

The most pronounced phenotype observed was the profound loss of MAIT cells in the absence of RelA, reminiscent of its impact on NK1.1^−^ to NK1.1^+^ NKT cell maturation,^23^, ^25^ and to a lesser extent, γδ17 T‐cell differentiation.^39^ Notably, RelA‐deficient MAIT cells retained the capacity to expand in response to cognate antigen *ex vivo*, suggesting RelA is not required for TCR‐mediated activation in these cells. This dissociation between developmental and functional competence suggests that RelA is not required for response to cognate antigen in the periphery, suggesting RelA may alternatively be activated by non‐TCR signals during thymic development. Given unconventional T cells are poised to be activated by both TCR and non‐TCR signals,^47^, ^48^ the loss of RelA may impair MAIT cell responses to infection via cytokine or inherent pathogen recognition signals even with intact TCR signaling. Supporting this, IL‐18 signaling, a known activator of the canonical NF‐κB pathway, has been implicated in MAIT cell development. Loss of IL‐18 impaired thymic MAIT cell numbers and progression to stage 3 thymic development,^9^ similar to the loss of RelA, although the absence of IL‐18Rα did not recapitulate this result.^9^ Despite the reduced MAIT1 population in RelA‐deficient mice, as defined by CD319 expression, IFNγ production was still detected, supporting the notion of a selective requirement for RelA in subset differentiation rather than effector function.

It is also possible that the immature phenotype of MAIT cells observed in RelA‐deficient mice reflects altered thymic retention and recirculation of mature cells.^49^ This would mirror the dual role c‐Rel plays in Treg development and retention/recirculation to the thymus.^50^ The inability of MAIT and NKT cells that do develop in RelA‐deficient mice to fill their niche in the periphery suggests that RelA also regulates the size of their shared peripheral niche, although this may be an indirect consequence of lower thymic production of these cells.^44^ While the signals that regulate the unconventional T‐cell niche have not been established, emerging evidence points to a gut‐thymus axis modulating MAIT cell development via microbial signals.^13^ The γc cytokines^51^ and/or IL‐1 family cytokines,^52^, ^53^ acting alone or secondary to microbial exposure, may equally play a role in shaping the unconventional T‐cell pool via RelA signaling.

The E3 ubiquitin ligases Hoip and Hoil, and the adaptor protein Sharpin, form the LUBAC complex and are associated with upstream signaling via NEMO leading to canonical NF‐κB activation.^35^, ^36^, ^37^ Conditional deletion of *Hoip* or *Hoil* in T cells leads to impaired MAIT cell development, reminiscent of RelA deficiency. In contrast, the loss‐of‐function mutation cpdm in *Sharpin* does not appear to significantly alter MAIT cell development or frequencies in peripheral lymphoid organs, consistent with observations in other T‐cell lineages.^54^ In line with this, the loss of the ligases, Hoil and Hoip, results in a complete loss of LUBAC signaling in MAIT cells, whereas loss of Sharpin only destabilizes/partially abrogates the complex.^55^, ^56^ LUBAC integrates signals from multiple immune receptors including TCR, IL‐1 receptors, Toll‐like receptors and TNFRSF members (TNFR, CD40 etc.), all of which have been implicated in MAIT cell development.^37^, ^57^, ^58^, ^59^ These findings support a model in which LUBAC‐mediated linear ubiquitination promotes MAIT cell development through activation of the RelA‐dependent canonical NF‐κB pathway in response to cytokine and/or microbial cues. More generally, our data fit a wider model whereby different canonical NF‐κB members distinctly control effector‐like T‐cell development or function, dependent on the anti‐ or pro‐inflammatory nature of the developing subset. For example, c‐Rel is the transcription factor most involved in the development of anti‐inflammatory thymic‐derived (t)Treg cells, whereas RelA is associated with Treg (anti‐inflammatory) effector function.^20^, ^60^, ^61^, ^62^, ^63^, ^64^, ^65^ Conversely, here we show RelA is more important for MAIT cell development than c‐Rel. Similarly, RelA is essential for the development of other (pro)‐inflammatory immune subsets, NKT cells^22^, ^23^, ^38^ and GC‐derived plasma cells,^42^ whereas c‐Rel is associated with the proper function of NKT cells,^23^ ILC2 cells,^66^ and B‐cell class switching.^67^ LUBAC signaling appears to promote NF‐κB signals in the development of unconventional T cells, and the development and homeostasis of Tregs,^54^ without the dichotomy displayed by individual NF‐κB transcription factors in differing cell types. Together with NF‐κB–independent LUBAC activity,^54^ this implies that LUBAC signaling is agnostic of the dominant NF‐κB family member and rather, re‐enforces the established transcriptional program. The distinct patterns of NF‐κB proteins controlling effector‐like lymphocyte development and function independently may offer a therapeutic strategy targeting development and/or function of inflammatory or regulatory populations via individual transcription factors.

In summary, we demonstrate a nonredundant, T‐cell‐intrinsic role for the classical NF‐κB pathway transcription factor RelA in MAIT cell thymic development, and to a lesser degree, the alternative pathway component NF‐κB2. The requirement for Hoip and Hoil in MAIT development, in parallel with RelA, underscores a role for both LUBAC and NF‐κB signaling governing the development of this unconventional T‐cell lineage. Along with previously implicated transcription factors such as PLZF and SATB1, NF‐κB RelA forms part of an important transcriptional program essential for establishing the effector‐like identity of MAIT cells during thymic development.

## METHODS

### Mice

The following mouse strains were maintained on an inbred Ly5.2 C57BL/6 background (> 10 generation backcross). Mice were age and sex matched within experiments. Generation and maintenance of *Rela* ^*+/−*^ (ref.^68^) and *cRel* ^−/−^ mice^41^ have been described previously. *Rela* ^*wt/fl*^ *Lck* ^*cre*^ mice that carry a Cre transgene expressed under the control of the *Lck* promoter (*Lck* ^*cre*^) and are heterozygous for floxed *Rela*,^69^ were then mated to generate litter‐matched mice homozygous for the wild‐type (*Rela* ^*wt/wt*^ *Lck* ^*cre*^) or floxed (*Rela* ^*fl/fl*^ *Lck* ^*cre*^) *Rela* genes. This strain has previously been crossed onto a *Foxp3* ^*rfp*^ (ref.^70^) background, although the *Foxp3* ^*rfp*^ transgene should not interfere with MAIT or NKT cell development. The experiments outlined in this study used age and sex‐matched mice bred in specific pathogen‐free facilities at Monash Animal Research Platform (Monash University, Clayton).

*Nfkb1* ^*−/−*^ mice^71^ were originally generated on a mixed C57BL/6x129SV background, but had been backcrossed onto a C57BL/6 background for > 10 generations before commencement of the studies presented here. *Nfkb2* ^*−/−*^ mice^72^ were maintained on a C57BL/6 background for > 10 generations. The *Rnf31*(*Hoip*)‐floxed,^73^ *Rbck1*(*Hoil1*)^55^ strains were crossed to *CD4* ^*cre*^ (ref.^54^) and maintained on a C57BL/6 background. *Sharpin* ^*cpdm*^ mice^56^ on a C57BL/Ka background were obtained from Jackson Laboratories and were backcrossed onto the C57BL/6 background twice. Mice were housed at The Walter and Eliza Hall Institute of Medical Research under specific pathogen‐free conditions.

The genotypes of the different mouse strains were determined by PCR screening of tail or ear clip samples. Ly5.1 (C57BL/6 *Ptprc* ^*a*^) mice were bred at Monash Animal Research Platform or obtained from the Walter and Eliza Hall Institute. All mouse experiments were performed in accordance with the animal ethics guidelines of the National Health and Medical Research Council of Australia with the approval of the Alfred Medical Research and Education Precinct, Monash Animal Research Platform, Walter and Eliza Hall Institute, University of Queensland, or Australian National University animal ethics committees.

### Lymphocyte isolation

Spleen and lymph node cells were isolated and prepared as previously described.^61^ Lymphocytes in the liver were isolated by mechanical digestion followed by density gradient enrichment. Briefly, liver tissue was pushed through a 70 μM filter, and the resulting cell suspension pelleted by centrifugation for 4 min at 400 × g, at room temperature. These cells were resuspended in 33% Percoll (GE, Chicago, USA) gradient by centrifugation for 12 min at 2000 rpm without breaks, at room temperature, and red cells lysed in RBC lysis buffer (Sigma‐Aldrich, Taufkirchen, Germany). Lymphocytes were collected and washed in Hank's balanced salt solution (HBSS) prior to performing antibody stains.

### Flow cytometry

Cells were first stained with PE or BV421‐conjugated MR1‐5‐OP‐RU or MR1‐Ac‐6‐FP tetramers alongside viability dye 7‐aminoactinomycin D (7‐AAD; Sigma‐Aldrich), LiveDead aqua or LiveDead near infrared (Invitrogen, Waltham, USA). Cells were then washed and blocked with Avidin/Biotin block (Dako/Agilent, Santa Clara, USA) and stained with the cell surface antibodies (Table 1) and CD1d‐α‐GalCer tetramers conjugated to BV421 or PE at 4°C in the dark for 30 min. Cells were then analyzed using a LSR Fortessa (Becton Dickinson (BD) Biosciences, Franklin Lakes, USA). Data were analyzed using FlowJo software (BD). After excluding dead cells and doublets by electronic gating, B220^−^ lymphocytes were then gated on for further analysis (Supplementary figure 3).

**Table 1:** Antibodies used for flow cytometry.

Target	Clone	Fluorochrome	Manufacturer
B220	RA3‐6B2	BUV496	BD
TCRβ	H57‐597	APC‐Cy7	BioLegend
TCRβ	H57‐597	PE‐Cy7	eBioscience
CD4	RM4‐5	BUV395	BD
CD8α	53–6.7	BUV805	BD
NK1.1	PK136	PE‐Cy7	BD
CD44	IM7	AF700	BD
CD24	M1/69	BUV737	BD
CD319	4G2	APC	BioLegend
CD138	281–2	BB515	BD
CD25	PC61.5	BV785	BD
IFNγ	XMG1.2	PE	BD
IL‐17	TC11‐18H10	AF647	BD
NK1.1	PK136	FITC	BD
CD45	30‐F11	BV785	BD
CD8β	H35‐17.2	AF488	eBioscience
TCRγδ	GL3	APC	BioLegend
CD8α	53–6.7	APC‐Cy7	BD
CD4	RM4‐5	BV605	BioLegend
B220	RA3‐6B2	BV786	BD
CD45.1	A20	APC	eBioscience
CD45.2	104	FITC	BD

For cytokine staining, splenocyte cell suspensions were stimulated with PMA (10 ng mL^−1^; Sigma‐Aldrich) and ionomycin (1 μg mL^−1^; Sigma‐Aldrich) in the presence of GolgiStop (BD Biosciences) for 4 h at 37°C. Cells were then washed with FACS buffer and stained with cell surface markers. Prior to staining for intracellular cytokines, cells were then fixed and permeabilized using the BD Cytofix/Cytoperm kit (BD Biosciences) in accordance with the manufacturer's instructions. Cells were then stained for anti‐IL‐17A and anti‐IFNγ for flow cytometric analysis.

### Reagents

5‐OP‐RU was synthesized as a stable solution in DMSO, as previously described.^74^, ^75^ Tetramers of mouse MR1‐5‐OP‐RU and MR1‐Ac‐6‐FP tetramers were generated as previously described.^76^, ^77^ Biotinylated MR1‐5‐OP‐RU and MR1‐Ac‐6‐FP monomers were tetramerized using streptavidin conjugated to PE (SAv‐PE; Invitrogen Molecular Probes).

Soluble mouse CD1d/β2m protein was produced as previously described.^78^ Purified CD1d–biotin was loaded with the α‐GalCer analog, PBS‐44, a gift of P. Savage (Brigham Young University) at room temperature overnight at a 6:1 (lipid:protein) molar ratio. CD1d loaded with PBS‐44, hereby referred to as CD1d–αGalCer, was tetramerized with streptavidin conjugated to Brilliant Violet 421 (SAv‐BV421; BioLegend, San Diego, USA).

### Generating fetal liver hematopoietic stem cell (HSC) chimeras

Fetal livers were harvested from E13 embryos generated by time mating heterozygous (*Rela* ^*+/−*^) mice.^60^ Single cell suspensions (~10^6^ cells) of *Rela* ^*+/+*^ or *Rela* ^*−/−*^ E13 fetal liver derived hematopoietic cells were intravenously injected into irradiated (2 × 4.5 Gy) C57BL/6J CD45.1^+^ mice. HSC chimeras were given acidified drinking water containing antibiotics for 6 weeks following HSC engraftment and were used for experiments at a minimum of 10 weeks post‐transplantation. Chimerism was > 95% as measured by staining circulating B cells with anti‐CD45.2 and anti‐CD45.1 antibodies.

### Tissue culture

For 5‐OP‐RU stimulation assays, splenocytes were isolated by mechanical digestion followed by Histopaque (Sigma‐Aldrich) density gradient. Single cell suspension of enriched lymphocytes at 5 × 10^6^ mL^−1^ were plated into a 24‐well TC plate with 100 nM 5‐OP‐RU for 7 days. Cells were harvested and examined for MAIT cell expansion by flow cytometry.

### Statistics

Sample sizes were determined by prior experience.^23^ Given sample sizes, nonparametric statistical tests were used, and where necessary, corrected for multiple comparisons. Difference in cell frequencies between matched groups were analyzed by the Mann–Whitney *U* test or Kruskal–Wallis test with Dunn's correction for multiple comparisons. Antigen stimulation was assessed by Wilcoxon matched pairs signed rank test with Holm‐Šidák correction for multiple comparisons. Where breeding was performed as het × het matings, controls were taken from matched littermates; otherwise, age‐ and sex‐matched controls were obtained from the same facility. Graphs depict median ± IQR. Each data point represents a single individual biological replicate. Statistical analyses were performed in GraphPad Prism v10.4.2 (San Diego, USA).

## AUTHOR CONTRIBUTIONS

Conceptualization, SG and DIG; methodology, TSF, HFK, SG, and DIG; investigation, TSF, HFK, RG, DNJ, SS, HJN, CET and LAOR; resources, RT, DHDG, JYWM, DPF, VLB, CZ, LAOR and SG; writing – original draft, TSF, HFK, SG and DIG; writing – reviewing and editing, TSF, HFK, SG and DIG; supervision, SG and DIG; funding acquisition, DIG.

## CONFLICT OF INTEREST

The authors declare no competing interests.

## Supporting information


Supplementary figure 1
Supplementary figure 2
Supplementary figure 3

## Data Availability

The data that support the findings of this study are available from the corresponding author upon reasonable request.
