# Supplementary material for: The NF‐κB transcription factor RelA directs mucosal‐associated invariant T‐cell development
Source: Immunol Cell Biol. 2026 Feb 23;104(4):329–44. doi: 10.1111/imcb.70096 (PMC13071142; doi:10.1111/imcb.70096)
Supplement: Supplementary file 1 — Supplementary figure 1 Supplementary figure 2 Supplementary figure 3 [file IMCB-104-329-s001.docx]

Supporting information for

The NF-κB transcription factor RelA directs mucosal-associated invariant T cell development

Thomas S Fulford^1,2,^*, Hui-Fern Koay^1,^*, Raelene Grumont^2^, Darryl N Johnson^1,†^, Sebastian Scheer^2,‡^, Hendrik J Nel^3^, Ranjeny Thomas^3^, Jeffrey YW Mak^4^, David P Fairlie^4^, Charis E Teh^5,6^, Daniel HD Gray^5,6^, Vanessa L Bryant^5,6,7^, Colby Zaph^2^, Lorraine A O’Reilly^5,6^, Steven Gerondakis^2,#^, Dale I Godfrey^1,#^

1. Department of Microbiology & Immunology at the Peter Doherty Institute for Infection and Immunity, University of Melbourne, Parkville, Victoria, 3010, Australia
2. Biomolecular Discovery Institute, Department of Biochemistry and Molecular Biology, Monash University, Clayton, Victoria, 3800, Australia
3. Frazer Institute, University of Queensland, Woolloongabba, Queensland, 4102, Australia
4. Institute for Molecular Bioscience, University of Queensland, Brisbane, Queensland 4072, Australia
5. The Walter and Eliza Hall Institute of Medical Research, Parkville, Victoria, 3052, Australia
6. Department of Medical Biology, The University of Melbourne, Melbourne, Victoria 3052, Australia
7. Clinical Immunology and Allergy Department, Royal Melbourne Hospital, Melbourne, Victoria, 3050, Australia

^*^ These authors contributed equally

^†^ Current address: Materials Characterisation and Fabrication Platform, Department of Chemical Engineering, University of Melbourne, Parkville, Victoria, 3010, Australia.

^‡^ Current address: Department of Infection and Immunity, Luxembourg Institute of Health, Esch-sur-Alzette, Luxembourg

^#^ These authors contributed equally

Correspondence to: [godfrey@unimelb.edu.au](mailto:godfrey@unimelb.edu.au); [thomas.fulford@unimelb.edu.au](mailto:thomas.fulford@unimelb.edu.au); [hf.koay@unimelb.edu.au](mailto:hf.koay@unimelb.edu.au)

# Supplementary Figures

**Supplementary figure 1. Thymic development of NKT cells is regulated by NF-κB signalling.** Thymocytes from **(a)** *Nfkb1^+/+^* or *Nfkb1^–/–^*, **(b)** *Rel^+/+^* or *Rel^–/–^*, **(c)** *Lck^cre^Rela^wt/wt^* or *Lck^cre^Rela^fl/fl^*, or **(d)** *Nfkb2^+/-^* or *Nfkb2^–/–^* mice were stained with CD1d-αGalCer tetramer-BV421 and anti-TCRβ-APC-Cy7 and analysed for MAIT cell frequency by flow cytometry. Graphs depict median ± IQR. ns – non-significant; * *P* < 0.05 by the Mann-Whitney *U-*test. *N* = 5 (*Nfkb1^+/+^, Nfkb2^+/+^, Nfkb2^–/–^*), 11 (*Nfkb1^–/–^*), 6 (*Rel^+/+^, Rel^–/–^*), 7 (*Rela^wt/wt^*), or 8 *(Rela^fl/fl^*) biological replicates from ≥ 2 experiments each. Each data point represents an individual biological replicate.

**Supplementary figure 2. Haematopoietic stem cell (HSC) chimeras lacking RelA have reduced unconventional T cell frequencies.** Irradiated CD45.1^+^ mice were reconstituted with CD45.2^+^ *Rela^+/+^* or *Rela^–/–^* fetal livers. Ten weeks later, lymphocytes from the thymus, mesenteric lymph node (mLN) and liver of *Rela^+/+^* or *Rela^–/–^* HSC chimeras were stained with **(a)** MR1-5-OP-RU tetramer-PE or **(b)** CD1d-αGalCer tetramer-BV421 and anti-TCRβ-APC-Cy7, and donor CD45.2^+^ cells analysed for MAIT cell and NKT frequencies by flow cytometry. Graphs depict median ± IQR. ns – non-significant; * *P* < 0.05 by the Mann-Whitney *U-*test with Holm-Šidák correction for multiple comparisons. *N* = 4 (thymus), 5 (*Rela^+/+^* mLN), 6 (*Rela^–/–^* mLN), 7 (liver) biological replicates from 2 (thymus and mLN) or 3 (liver) experiments. Each data point represents an individual biological replicate.

**Supplementary figure 3. Flow cytometry gating strategy.** Lymphocytes were first gated based on their forward scatter and side scatter profile. After excluding doublets by forward scatter height and width and dead cells with LiveDead aqua, B cells and autofluorescent cells were excluded with B220 and an empty channel (B670/30) respectively. B220^–^ lymphocytes were then gated on for further analysis.
